# The Administration of Anæsthetics

**Published:** 1898-08

**Authors:** 


### Abstracts and Translations.

### THE ADMINISTRATION OF ANÆSTHETICS.

Upon this topic, one of cardinal importance to every practitioner of medicine, there have lately appeared in the London *Lancet* three lectures delivered by Frederic Hewitt, based generally on a wide experience as an anæsthetist, particularly on six thousand six hundred and fifty-seven administrations of anæsthetics conducted at the London Hospital during the year 1897. On the first of that year Hewitt adopted a system of recording the cases of anæsthesia, providing books and special tabular forms so that every instance of the administration of the narcotic in any part of the hospital could be properly noted. It is interesting to observe that out of the total number of cases nearly three thousand were of ether and more than one thousand of nitrous oxide. Chloroform was relied on solely in six hundred and seventy-seven cases, the A. C. E. mixture in five hundred and ten, ether followed by chloroform in two hundred and ninety-three, nitrous oxide and oxygen in two hundred and forty, and other successions in the majority of the cases unaccounted for. It will be noted that Hewitt has not confined himself to any particular method, but has had a sufficient clinical experience to speak with authority upon nearly all the combinations in common use.

As to chloroform, he states that within a few years it was the anæsthetic in routine use, but that confidence in it has been shaken by experience. He particularly warns against the employment of certain recently devised chloroform inhalers. He states that for giving chloroform in the ordinary routine way there is nothing better than Skinner's mask.

Referring to the general effect of chloroform as an anæsthetic, Hewitt states that, other things being equal, the stronger the patient the greater the trouble in giving the anæsthetic. Deaths from chloroform are the most common in the middle period of life, when men are most vigorous, and more men die than women under this anæsthetic. The drug is most lethal during the early stage of its administration, and a very large proportion of the accidents have occurred in connection with minor operations, which are most common in vigorous subjects. The explanation of this is dependent upon the fact that during the transient, very imperfect anæsthesia, the muscular system is thrown into a condition of spasm, most marked in the most powerfully developed.

Hewitt states that there is much to be said in favor of the use of ether and chloroform in succession. The stage of rigidity and excitement, which is the dangerous stage in chloroform, is safely passed over under the stimulant effects of ether. Having secured a proper degree of ether anæsthesia, chloroform may be substituted, and there will result a better type of chloroform anæsthesia than if this drug had been given from the beginning.

Hewitt has adopted this principal for several years and is able to use chloroform without the occurrence of those untoward symptoms which are bound to occasionally arise when this agent is given from the commencement of the administration. He regards this development in our methods as one of the most important of recent years. It is especially indicated when ether causes cough, embarrassed breathing, or the secretion of much mucus, or when the operation is likely to be a protracted one. Some practice is needed to know when and how to effect the change. The rule to follow is, there should always be some evidence of the patient having emerged from ether anæsthesia when the chloroform is applied. As a rule, the conjunctival reflex should be present when the change is effected, and in abdominal operations this change should be made before the operation is begun. Very little chloroform will be required to keep up the proper degree of unconsciousness.

As a result of a very large experience Hewitt uses much less ether and much more chloroform than formerly. He holds that it is as safe to keep up the anæsthesia of chloroform as that of ether, and that the risk of subsequent bronchial and pulmonary complications is undoubtedly reduced. He has often anæsthetized according to this procedure the patients in the sitting posture, and in not a single instance has he been compelled to place them horizontally.

By his arrangement of cylinders he is able to administer nitrous oxide and oxygen in combination and to keep up the anæsthesia for an almost indefinite time. The method is the safest at present known and is devoid of after-effects. The anæsthesia is not so deep as that of other agents commonly employed; the muscular system is not so completely relaxed; nor is it possible always to completely abolish reflexes. He is not satisfied with it in severe surgical cases. His best results have been with rather debilitated women or children. Robust and vigorous male adults, especially those of alcoholic habit or excessive smokers, are not good subjects. In one case of breast excision, in which the anæsthesia was kept up for thirty-five minutes, a good deal of vomiting followed.

Hewitt's observations concerning the use of nitrous oxide before ether are especially serviceable. This plan of anæsthetizing is at times inapplicable and unsuccessful. Cases are seen in which there has been a narrow escape from fatal asphyxia. Muscular men of middle life who have become rather obese should never be anæsthetized by this method. If it is employed only a small quantity of nitrous oxide should be used, and ether should be gradually added to it. The plan of administering a full dose of nitrous oxide and changing to ether is, however, very useful in children and in women, provided that there are no contraindications to either in the patients.

The use of nitrous oxide, ether, and chloroform in succession is warmly commended. The initial anæsthetic, nitrous oxide, rapidly destroys consciousness and prevents struggling. The intermediate anæsthetic, ether, is useful because the circulation will remain unimpaired by the strain imposed upon it should rigidity or suspended breathing arise before deep anæsthesia, and because the stimulant effect produced by the ether lasts for a considerable time while chloroform is being given. Chloroform is administered because of the quiet and deep anæsthesia which it produces, because of its great convenience, and because of the rarity with which bronchial and pulmonary after-effects are met with after its use. Hewitt states that, given no special contraindications, there is no better plan of anæsthesia than this.

The A. C. E. mixture he commends for middle-aged and powerfully built men, with double chins and thick necks. It is first dropped upon an open Skinner mask; after a couple of minutes a Rendle mask with more of the mixture upon it may be substituted; and immediately any rigidity begins to show itself the Rendle mask may be exchanged for an Ormsby inhaler charged with ether. By this plan he has been enabled to successfully anæsthetize without any difficulty many such subjects.

Of the total number of cases anæsthetized by Hewitt, there were thirteen which exhibited threatening symptoms. The four factors giving rise to dangerous symptoms are, he states, the anæsthetic itself, the state of the patient, the posture of the patient, and the surgical operation.

In the first case which exhibited dangerous symptoms, breathing stopped before the patient was thoroughly anæsthetized. As the patient was greatly cyanosed, ten ounces of blood were drawn, and the breathing immediately commenced. It is very common, just before anæsthesia is established, for the breathing to become temporarily suspended. This is due to acts of swallowing, which are more tardily performed than when consciousness is intact, thus closing the glottis. In addition there may be general muscular spasm. In the vast majority of cases this impaired breathing comes on just before stertor and passes off spontaneously, or may be made to do so by removing the inhaler, rubbing the lips with a towel, or pushing the lower jaw forward. In more obstinate cases, in which the jaws are clinched and the neck muscles are rigidly contracted, it may be necessary to separate the teeth and to pass the finger to the back of the pharynx, when breathing will recommence. Unless this condition is remedied it may pass on to a dangerous or even fatal degree of asphyxia.

Of the six thousand six hundred and fifty-seven cases of anæsthesia, there were only three in which the anæsthetic could in any way be held responsible for the fatal issue, and only one in which the anæsthetic was the sole factor.

In a case of appendicitis following typhoid fever the patient apparently died of bronchopneumonia thirty-four hours after operation. Hewitt holds that the chances of pulmonary complications would have been less if chloroform or the A. C. E. mixture had been given, and that from this experience it is fair to infer that the unusual cyanosis met with during the administration might with advantage have been taken to indicate that a change from ether to chloroform should have been effected.

The immediately fatal case occurred under chloroform and during the period of preliminary excitement.

As to the gastric after-effects of anæsthetics other than nitrous oxide, in two hundred and seventy-five cases in which this was noticed there were none in one hundred and nine, slight in one hundred and nine, moderately bad in thirty-five, and severe in twenty-two. Calculating on a percentage basis, it is noted that thirty-six per cent. of the ether cases had no after-effects; while thirty-two per cent. of the ether cases followed by chloroform were similarly fortunate; seven and two-tenths per cent. exhibited severe after-effects after ether. Patients are more apt to suffer from slight after-effects when ether has been used, but the chances of protracted vomiting are greater when chloroform has been administered. There can be no doubt that abstinence from food for at least four hours before operation should be enforced. Clear soup or beef tea is the best form of nourishment before operation. The practice of giving a patient beef tea three hours before operation is open to objection. The stomach should not only be emptied, but digestion should have been finished for some little time.

A tabulation shows that the severity of the after-effects is markedly influenced by the continuance of the anæsthetization. As to the prevention of the gastric after-effects, in addition to previous regulation of the diet it is essential that the bowels should be thoroughly evacuated; this is accomplished by administering a purgative on the night before the operation and an enema on the morning itself. The patient should be rendered anæsthetic as rapidly as possible; a deep anæsthesia, free from swallowing movements, should be maintained. The head should be kept to the side for the escape of mucus and saliva, and the mouth should be frequently wiped out. The patient when in bed should be turned well upon his side and the bed should not be moved, and the room should be kept quiet. No nourishment should be given until the patient himself asks for it.

If nausea, retching, or vomiting be present the patient should be given at intervals two or three ounces of very hot water to drink. The taste of ether is best overcome by moistening the lips with lemon juice. If there appears to be a neurotic element present, enemata of twenty grains of bromide of potassium in two ounces of water will often answer well. The inhalation of vinegar from a towel is very useful in arresting vomiting, as is also the application of mustard leaf to the epigastrium.

This communication of Hewitt's is extremely important, as it is founded upon the careful, minute, detailed observation of an accomplished and widely experienced anæsthetist. The diversity of his methods and the multiplicity of his apparatus do not commend themselves to an American surgeon. One important lesson, however, taught by his paper is that there are merits in ether, chloroform, and nitrous oxide; that each has its appropriate place in surgery; and that in place of the slavish adherence to routine it would be wise to judiciously select in accordance with the condition and temperament of the patient. His practical observations upon certain difficulties that arise during anæsthetization, his strictures concerning the employment of chloroform and nitrous oxide in the robust, are useful. His reasons for substituting ether by chloroform will scarcely be conclusive to American surgeons, save in exceptional cases,—*i.e.*, those with a tendency towards pulmonary congestion. Under such circumstances the following of Hewitt's plan may well be commended.—*The Therapeutic Gazette.*

